# Supplementary material for: Health Technology Assessment Development in Vietnam: A Qualitative Study of Current Progress, Barriers, Facilitators, and Future Strategies
Source: Int J Environ Res Public Health. 2021 Aug 22;18(16):8846. doi: 10.3390/ijerph18168846 (PMC8392551; doi:10.3390/ijerph18168846)
Supplement: Supplementary file 1 [file ijerph-18-08846-s001.zip › ijerph-1307131-supplementary/ijerph-1307131.pdf]

Table S1. Semi structured interview questionnaire

1.Explain information on purpose of interview and research project.

2. Get a consent form

3. Questions

1) Introduction

- Date of interview
- Age
- Gender
- Educational background? highest year of school completed?
- Please introduce yourself
- What are you working on in current job? And have you done work related health technology assessment or priority setting in health insurance reimbursement? If so, please explain in detail? Which position?
- What type of organization do you work in (Federal government, regional government, district government, research institute, multilateral, bilateral, private)? / What is your position?
- How many years in your current position? how many of work experience in public health do you have?
- What is your academic background related health?

2) Questions about current stage in HTA development in Vietnam

- Are you familiar with the concept of economic evaluation or health technology assessment?
- Have you (or your colleagues) had education or training in health economics? Who provided it? How much it was helpful?
- Are there any forms of network of HTA experts (government staffs, private, academia)?
- Is there any legal stage related to HTA?
- Is economic evaluation a formal requirement for health insurance reimbursement

currently?

- Does government have formal guidelines for HTA?
- Did you ever use an economic evaluation for decision-making?
- What is current government effort for developing HTA?
- Is there any research or project going on about priority setting process in Vietnam? (government or university?)
- Currently, who is responsible for allocative efficiency in healthcare (microscopically or macroscopically): the professionals or government?
- Could you explain current priority setting process (decision making process) in health reimbursement decision making?
- Currently. what are the most important criteria in priority setting in health insurance reimbursement or budget allocation?
- What do you think is the strengths or weaknesses of current system?

3) Barriers to HTA development and what should be done.

- Do you think economic evaluation should be a formal requirement in priority setting for reimbursement?
- What do you think is the biggest barrier for adoption of economic evaluation as formal criteria in priority setting? Why do you think it is a barrier?
- What do you think should be done to remove those barriers? At government level, organization level, and academia.
- What kind of efforts are being taken currently by government?
- What kind of education is most necessary?
- What topics of concern do you think can be potentially benefit from economic evidence?
- Do you think there should be an explicit cut-off point(threshold) for cost-effectiveness? or do you think there should be some range?
- If you prefer explicit cut-off point, how much do you think is appropriate for certain health technology to be cost-effective?
- Do you think the same threshold should be applied to all? If not, any thought?

(Explain Norway's case if necessary)

- Do you think economic evaluation should be used in medical equipment or other area?
- In university, what kind of research is being done in terms of economic evaluation?

4) Questions about economic evaluation in priority setting in health reimbursement

- Are you familiar with the concept of priority setting?
- Do you think it is ethical to refuse treatments for cost-effective reasons? Generally? or for specific product (or treatment?)
- Which effort is necessary for the priority setting process mechanism more systematic, fair, more responsive?
- Who do you think should be involved in priority setting of health insurance reimbursement? / Do you think citizen's opinion should be reflected in priority setting?

※ Are there anything you would like to add? That I have not asked about?
